# Depth of Response was Associated with Progression-Free Survival in Patients with Advanced Non-small Cell Lung Cancer treated with EGFR-TKI

**DOI:** 10.7150/jca.33450

**Published:** 2019-08-28

**Authors:** Yu-Tao Liu, Kai Zhang, Cheng-Cheng Li, Xing-Sheng Hu, Jun Jiang, Xue-Zhi Hao, Yan Wang, Jun-Ling Li, Pu-Yuan Xing, Sheng Yang, Xin Zhang, Guo-Qiang Wang, Shang-Li Cai, Yuan-Kai Shi

**Affiliations:** 1 Department of Medical Oncology, Beijing Key Laboratory of Clinical Study on Anticancer Molecular Targeted Drugs, National Cancer Center/ National Clinical Research Center for Cancer/ Cancer Hospital, Chinese Academy of Medical Sciences & Peking Union Medical College, Beijing, P.R. China; 2 Cancer Center of Union Hospital, Tongji Medical College, Huazhong University of Science & Technology, Wuhan, P.R. China; 3 The Medical Department, 3D Medicines Inc, Shanghai, P.R. China; 4 Department of radiology, Beijing Key Laboratory of Clinical Study on Anticancer Molecular Targeted Drugs, National Cancer Center/ National Clinical Research Center for Cancer/ Cancer Hospital, Chinese Academy of Medical Sciences & Peking Union Medical College, Beijing, P.R. China

**Keywords:** DepOR, NSCLC, EGFR-TKI, targeted therapy

## Abstract

**Background**: Response Evaluation Criteria in Solid Tumors (RECIST) has been widely utilized to evaluate new therapeutic strategies in cancer. However, RECIST fails to assess the heterogeneity of response in highly active therapies. Depth of response (DepOR), defined as the maximum percentage change in tumor size compared with baseline, may provide a new strategy to evaluate disease response. In the present study, we studied the association between DepOR and progression-free survival (PFS) in patients with advanced non-small cell lung cancer (NSCLC) treated with epidermal growth factor receptor tyrosine kinase inhibitor (EGFR-TKI).

**Methods**: Advanced NSCLC patients harboring EGFR driver mutation (L858R or exon 19 deletion) treated with EGFR-TKI from August 2014 to July 2017 from two sites were retrospetively collected for analysis. Patients were divided into four groups by DepOR (Q1 = 1-25%, Q2 = 26-50%, Q3 = 51-75%, Q4 = 76-100%). Kaplan-Meier curves were plotted for PFS against DepOR and the hazard ratio (HR) was determined through univariable and multivariable cox regression models.

**Results**: In total, 265 patients were included for analysis. The number of patients in Group Q1-Q4 were 91 (34.3%), 73 (27.5%), 65 (24.5%) and 36 (13.6%), respectively. A greater DepOR was significantly associated with a longer PFS (Log-rank *P*<0.0001). The HRs (95% CI) for PFS comparing patients with different DepOR status were 0.58 (0.42-0.80) for Q2, 0.49 (0.35-0.69) for Q3, and 0.33 (0.22-0.50) for Q4, all compared with patients in Q1. DepOR as a continuous variable was also associated with prolonged PFS (HR, 0.20; 95% CI, 0.13-0.33; P<0.001). Additionally, in the multivariable cox regression model, abnormal LDH, brain metastasis and male were found to be associated with worse PFS outcomes (P<0.05).

**Conclusion**: A greater DepOR is significantly associated with PFS benefit in advanced NSCLC treated with EGFR-TKI, suggesting that it may be a useful clinical outcome to evaluate the response of targeted therapy.

## Introduction

Response Evaluation Criteria in Solid Tumors (RECIST) has been widely utilized to evaluate new therapeutic strategies in cancer. Objective response rate (ORR) could be used as an effective surrogate clinical endpoint for overall survival (OS) to predict tumors response via an anatomical approach with a unidimensional measurement of tumor burden according to the RECIST^1^. However, RECIST fails to assess the heterogeneity of response in highly active therapies, or to discern earlier read-out of activity for new therapeutics, and it also showed some limitations in predicting OS in some targeted therapies^2^-^5^.

In ALEX trial, no significant difference was observed between ORR in non-small cell lung cancer (NSCLC) patients treated with alectinib versus crizotinib (83% vs 76%; p=0.09). However, patients treated with alectinib obtained dramatically prolonged progression-free survival (PFS) compared with those treated with crizotinib^3^. Another trial with afatinib, an epidermal growth factor receptor (EGFR) tyrosine kinase inhibitor (TKI), showed a higher ORR (70% vs 56%; p=0.0083) compared with gefitinib in NSCLC, however, such benefit was not observed for OS^5^. The above results suggested that alternative evaluation tools are needed to supplement RECIST with improved evaluation of clinical benefits in the NSCLC patients treated with targeted therapies.

The depth of response (DepOR), defined as the maximum percentage change in tumor size compared with baseline, has been indicated in several clinical trials to fetch up the insufficiency of RECIST in solid tumor evaluations. Increased DepOR were associated with a significantly longer post-progression survival (PPS) or OS in colorectal cancer patients treated with cetuximab or bevacizumab^6^-^8^. Choong-Kun Lee et al. further reported that DepOR could serve as a predictor for long-term outcome in advanced gastric cancer patients treated with trastuzumab^9^. The DepOR has also been investigated in the NSCLC patients treated with ALK inhibitor or anti-PD-1 antibody^10^. Altogether, the studies suggested DepOR to be an effective complement evaluation endpoint for PFS or OS.

Given the rapid development of targeted therapies in advanced NSCLC and the inadequacy of RECIST for monitoring treatment response, in this study, we aimed to provide a new strategy to assess disease response by evaluating the association between DepOR and PFS in NSCLC patients treated with EGFR-TKI.

## Materials and Methods

### Patients

A two-institution retrospective cohort analysis was performed at National Cancer Center/ Cancer Hospital of Chinese Academy of Medical Sciences and Cancer Center of Union Hospital, Tongji Medical College, Huazhong University of Science & Technology. Advanced NSCLC patients harboring EGFR driver mutation (L858R or exon 19 deletion mutation) treated with EGFR-TKI (gefitinib, icotinib and erlotinib) as first-line treatment from August 2014 to July 2017 were included in this study. The exclusion criteria was: (1) Patients with malignant tumors history (except for basal cell or in situ cervical cancer and completely resected intramucosal gastric cancer) other than lung cancer; (2) Patients ever treated with EGFR TKI for postoperative adjuvant treatment or harboring EGFR T790M primary mutation; (3) Incomplete electronic medical records; (4) Patients without evaluable target lesion.

### Data collection

For all patients, the following pretreatment demographic and clinical information were obtained from medical records: age, sex, TNM staging, pathology, EGFR mutation, smoking status, baseline lactic dehydrogenase (LDH), prior treatment, metastatic sites and combined therapy. Target lesion responses were assessed by two independent radiologists via computed tomography (CT) according to RECIST. DepOR was defined as maximum percent change in tumor size compared with baseline, patients were divided into four groups by maximal tumor shrinkage (Q1 = 1-25%, Q2 = 26-50%, Q3 = 51-75%, Q4 = 76-100%). PFS was defined as the interval from the start date of EGFR-TKI administration to the date of progression or death due to any cause.

### Statistical analysis

Continuous variables were expressed as means and standard deviations (SDs) if normally distributed or median (range) if not normally distributed. Categorical variables were expressed as number and percentages. Survival probabilities were estimated using the Kaplan-Meier method, and a log-rank test was used to evaluate differences between survival curves. A Cox proportional hazards regression model was used for univariate and multivariate analyses. In the multivariable cox regression model including variables with P value < 0.10 in the univariate cox regression. All P-values were two-sided, and P < 0.05 was considered statistically significant. Statistical analyses were performed with SPSS version 20.

## Results

### Patient demographics and characteristics

The baseline demographics and characteristics for the patients are listed in **Table 1**. In total, 265 patients were included for the analyses, with 124 (46.8%) from National Cancer Center/Cancer Hospital of Chinese Academy of Medical Sciences and 141 (53.2%) from Cancer Center of Union Hospital, Tongji Medical College, Huazhong University of Science & Technology. The median patient age was 61 (range 32-89) years, and 142 (53.6%) patients were male. 258 patients (97.4%) were adenocarcinoma, 256 (96.7%) patients had an Eastern Cooperative Oncology Group (ECOG) performance status (PS) of 0-1, and 157 (59.2%) patients were never smokers. Patients were divided into four groups acoording to the maximal tumor shrinkage (Q1 = 1-25%, Q2 = 26-50%, Q3 = 51-75%, Q4 =76-100%). The patient numbers for Group Q1-Q4 were 91 (34.3%), 73 (27.5%), 65 (24.5%), and 36 (13.6%), respectively. The patient demographics and disease characteristics were well-balanced across the quartiles for most variables except that patients in Group Q4 have a higher frequency of a normal lactate dehydrogenase level.

### Association between DepOR and PFS

**Figure 1:**
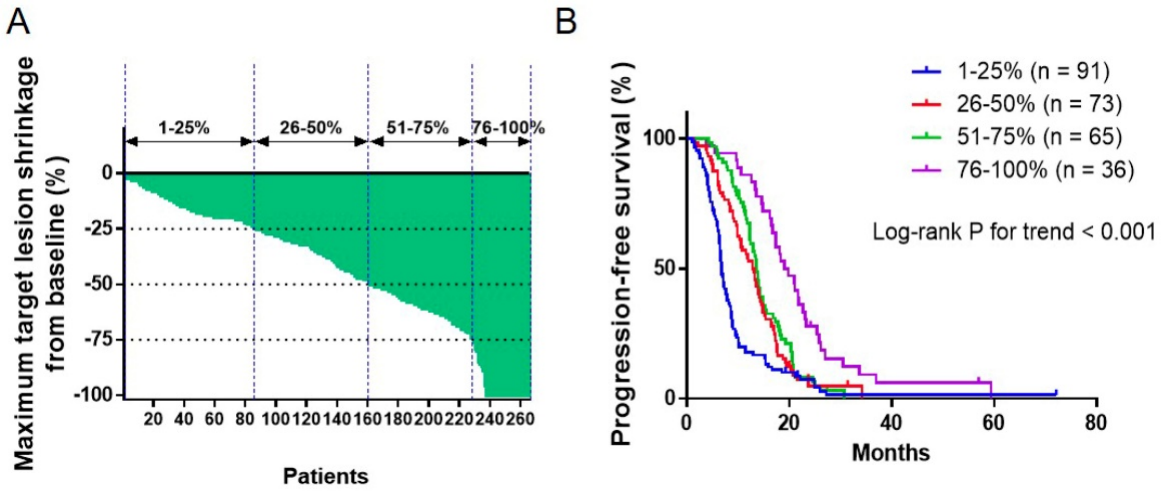
** Association between depth of response and progression-free survival**. (A) Waterfall plot depicting the maximum tumor shrinkage for each patient. (B) Kaplan-Meier survival curves of progression-free survival comparing patients with a maximum tumor shrinkage of 1-25%, 26-50%, 51-75% and 76-100%.

The association between DepOR and PFS was determined among patients in Groups Q1-Q4 that were divided upon the maximal tumor shrinkage as described above. The maximum tumor reduction for each patient was depicted in **Figure 1A**. The median PFS for patients from Groups Q1-Q4 were 6.8 months, 13.0 months, 13.7 months and 19.4 months, respectively (P<0.05). Kaplan-Meier survival curves were plotted for PFS against DepOR (**Figure 1B**), showing that a greater DepOR was significantly associated with a longer PFS (Log-rank *P* < 0.0001).

### Univariate and multivariate analysis of progression-free survial of patients treated with EGFR-TKI

In the univariate analysis, DepOR was significantly associated with PFS either as a rank variable (HR, 0.68; 95% CI, 0.60‑0.76; P<0.001, **Table 2**) or as a continous variable (HR, 0.20; 95% CI, 0.13-0.33; P < 0.001, **Table 2**). In addition, sex, liver metastasis, brain metastasis, surgery prior to the use of TKI, and LDH level were associated or tended to be associated with the progression-free survival. Variables with a P value <0.10 in the univariate anlaysis were subsequently included in the multivariate cox regression model, showing that sex, brain metastasis, LDH level, and DepOR remained to be significantly associated with progression-free survival (**Table 2**).

Moreover, patients in Q1 were used as a comparative cohort for further analysis. Superior PFS were observed for patients in Groups Q2-Q4 when compared with patients in Q1 (Q2, HR 0.58, 95% CI, 0.42-0.80, P<0.001; Q3, HR 0.49, 95% CI, 0.35-0.69, P<0.001; Q4: HR 0.33, 95% CI, 0.22-0.50, P<0.001; **Table 3**). After adjusting the potential confounding factors including LDH level (normal vs abnormal), brain metastasis, and sex, DepOR was still an independent prognositc factor for PFS with a HR of 0.57 (0.40-0.80, P<0.001) for Q2, 0.36 (0.25-0.52, P<0.001) for Q3 and 0.32 (0.20-0.51, P<0.001) for Q4 as compared with paitnets in Q1 (**Table 3**).

## Discussion

In the present study, we have evaluated the relationship between DepOR and PFS in NSCLC patients treated with EGFR-TKI. The results demonstrated that a greater DepOR is associated with PFS benefit from EGFR-TKI treatment. Besides, brain metastasis, abnormal LDH level, and male were associated with worse PFS.

OS is currently the golden standard for oncology clinical trial endpoints^11^, however, it is inefficient and time-consuming for clinical evaluation, thus finding a surrogated early endpoint is important for clinical assessment. ORR evaluated by RECIST criteria has been widely applied by clinicians. However, previous studies revealed that ORR may fail to capture the full benefit of treatment^11^ and discern earlier read-out of activity for new therapeutics. In addition, ORR outcome is not always consistent with PFS or OS in evaluating endpoints in oncology^3^,^4^. Under these circumstances, DepOR has been proposed as a complement factor for ORR to evaluate the clinical benefit^7^.

Investigations have been carried out in several solid tumors using DepOR as a clinical endpoint. In the phase III trial comparing cetuximab in combination with FOLFIRI as first-line treatments in colorectal cancer patients, an increased DepOR, while not ORR, was associated with a longer OS in patients treated with cetuximab plus FOLFIRI^6^. DepOR has also been studied in a post-hoc analysis of CRYSTAL and OPUS trials in colon cancer, in which a greater DepOR was demonstrated to be associated with longer OS and post-progression survival^8^. Another clinical trial showed that gastric cancer patients with a DepOR ≥ 45% obtained longer PFS and OS when treated with trastuzumab^9^. In fact, the satisfactory performance of DepOR as an evaluation factor has not only been shown for targeted therapies, but also for immunotherapy in NSCLC^10^. In addition, DepOR has been investigated in hematologic malignancies to evaluate the changes of M-protein in multiple myeloma and minimal residual disease in leukemia and myeloma^12^-^15^, implying the validity of DepOR as a complementary endpoint established for different interventions and different neoplasms.

In the present study, we demonstrated that a greater DepOR was significantly associated with a longer PFS. After adjustment of potential confounding factors including LDH level, brain metastasis, and sex, DepOR was still an independent prognositc factor for PFS. We have proved that DepOR could serve as an effective complementary clinical endpoint to evaluate the clinical benefit of EGFR TKI in patients with NSCLC.

Besides, we showed that an abnormal lactate dehydrogenase level was associated with worse PFS. LDH has been widely used as a classic inflammatory marker for lung cancer patients treated with chemotherapy or targeted therapies^16^-^18^. One previous study demonstrated that an abnormal LDH level might be a negative predictor of survival in NSCLC patients receiving immune checkpoint blockades^19^. However, our study revealed that the abnormal LDH was associated with shorter PFS in advanced NSCLC patients treated with EGFR-TKI, suggesting that LDH may be an independent prognosis indicator for NSCLC instead of a predictive biomarker for immunotherapy.

We also found that brain metastasis was a potential poor prognostic factor independent of the DepOR. Indeed, EGFR-TKIs administrated in this study, including gefitinib, icotinib and erlotinib, have been evaluated in patients with brain metastasis with a mild benefit^20^-^22^, probably because of their poor capability to penetrate the blood-brain barrier. For a majority of the NSCLC patients with central nervous system (CNS) metastasis, brain metastasis was the first site of treatment failure after initial response^23^, supporting the aforementioned blood-brain barrier hypothesis.

Our study has provided a new strategy to evaluate the clinical outcomes, however, there are still some limitations need to be considered. Firstly, this is a retrospective study that may yield selecting bias. Nevertheless, the patient demographics and disease characteristics were well-balanced across the quartiles for most variables. Secondly, the association between DepOR and OS was not analyzed due to the consideration of the changes of treatment for patients upon disease progression, which may introduce potential bias to the results. Thirdly, in our study, the association between DepOR and PFS was conducted only in patients with tumor shrinkage due to the limited number of patients with progressed disease upon EGFR-TKI. Last but not least, the ability of response assessment between DepOR and RECIST was not compared directly, a larger prospective study is warranted for further studies. In summary, we have demonstrated that DepOR was associated with better PFS either as a rank variable or a continuous variable, suggesting that the DepOR may be a supplement to RECIST to evaluate the treatment response in future clinical practice.

## Conclusion

The DepOR may be a useful clinical outcome to efficiently evaluate the response of EGFR-TKI in patients with NSCLC. More evidences are needed to understand how to use DepOR as an endpoint in clinical trials.

## Figures and Tables

**Table 1:** Baseline demographics and characteristics of all patients.

		All (n = 265)	1-25% (n = 91)	26-50% (n = 73)	51-75% (n = 65)	76-100% (n = 36)
Sex, n (%)						
	Male	142 (53.6)	60 (65.9)	34 (46.6)	32 (49.2)	16 (44.4)
	Female	123 (46.4)	31 (34.1)	39 (53.4)	33 (50.8)	20 (55.6)
Age						
	Median	61	61	60	59	63.5
	Range	32-89	42-89	32-78	32-82	38-82
Stage						
	IIIB	16 (6)	7 (7.7)	3 (4.1)	4 (6.2)	2 (5.6)
	IV	249 (94)	84 (92.3)	70 (95.9)	61 (93.8)	34 (94.4)
ECOG						
	0	12 (4.5)	5 (5.5)	1 (1.4)	4 (6.2)	2 (5.6)
	1	244 (92.1)	83 (91.2)	70 (95.9)	59 (90.8)	32 (88.9)
	2	7 (2.6)	3 (3.3)	1 (1.4)	1 (1.5)	2 (5.6)
	Unknown	2 (0.8)	0 (0)	1 (1.4)	1 (1.5)	0 (0)
Smoking						
	Current	43 (16.2)	18 (19.8)	12 (16.4)	9 (13.8)	4 (11.1)
	Previous	33 (12.5)	14 (15.4)	7 (9.6)	7 (10.8)	5 (13.9)
	Never	157 (59.2)	50 (54.9)	40 (54.8)	40 (61.5)	27 (75)
	Unknown	32 (12.1)	9 (9.9)	14 (19.2)	9 (13.8)	0 (0)
Pathology						
	adenocarcinoma	258 (97.4)	91 (100)	70 (95.9)	62 (95.4)	36 (100)
	squamous carcinoma	3 (1.1)	0 (0)	2 (2.7)	1 (1.5)	0 (0)
	adenosquamous carcinoma	4 (1.5)	1 (1.1)	1 (1.4)	2 (3.1)	0 (0)
EGFR mutation						
	19 del	134 (50.6)	39 (42.9)	32 (43.8)	41 (63.1)	22 (61.1)
	L858R	129 (48.7)	52 (57.1)	41 (56.2)	24 (36.9)	14 (38.9)
EGFR-TKI						
	geftinib	106 (40.0)	44 (48.4)	21 (28.8)	26 (40.0)	15 (41.7)
	icotinib	125 (47.2)	34 (37.4)	43 (58.9)	31 (47.7)	17 (47.2)
	erlotinib	34 (12.8)	13 (14.3)	9 (12.3)	8 (12.3)	4 (11.1)
Metastasis						
	Liver	52 (19.6)	18 (19.8)	17 (23.3)	11 (16.9)	6 (16.7)
	Intrathoratic	194 (73.2)	66 (72.5)	59 (80.8)	41 (63.1)	28 (77.8)
	Brain	63 (23.8)	28 (30.8)	12 (16.4)	18 (27.7)	5 (13.9)
	Bone	68 (25.7)	25 (27.5)	22 (30.1)	14 (21.5)	7 (19.4)
Surgery		67 (25.3)	20 (22)	14 (19.2)	16 (24.6)	17 (47.2)
LDH						
	Normal	165 (62.3)	46 (50.5)	49 (67.1)	39 (60.0)	31 (86.1)
	Abnormal	80 (30.2)	40 (44.0)	17 (23.3)	20 (30.8)	3 (8.3)
	Unknown	20 (7.5)	5 (5.5)	7 (9.6)	6 (9.2)	2 (5.6)

**Table 2:** Univariate and multivariate analyses of progression-free survival.

		Univariate analysis				Multivariate analysis		
		HR	95% CI	P		HR	95% CI	P
Sex								
	Male vs female	1.27	0.99-1.62	0.06		1.34	1.02-1.75	0.04
Age								
	≥65 vs <65	1.1	0.85-1.43	0.46				
Stage								
	IV vs IIIB	0.92	0.54-1.55	0.74				
EGFR driver mutation								
	L858R vs 19del	1.07	0.83-1.37	0.6				
Metastasis								
	Yes vs no	1.04	0.60-1.83	0.88				
Intrathoratic metastasis								
	Yes vs no	0.83	0.63-1.10	0.19				
Liver metastatic								
	Yes vs no	1.41	1.03-1.93	0.03		1.2	0.85-1.70	0.29
Brain metastatic								
	Yes vs no	1.83	1.37-2.46	<0.001		1.7	1.23-2.35	0.001
bone_metastatic								
	Yes vs no	1.22	0.92-1.62	0.17				
Surgery								
	Yes vs no	0.68	0.50-0.91	0.008		0.98	0.71-1.34	0.89
dNLR								
	>3 vs ≤3	1.14	0.62-2.11	0.67				
Lines of treatment		0.96	0.74-1.24	0.74				
DepOR (rank variable)		0.68	0.60-0.76	<0.001		0.62	0.54-0.71	<0.001
DepOR (continuous variable)		0.20	0.13-0.33	<0.001				
LDH								
	Abnoraml vs normal	2.9	2.17-3.86	<0.001		2.82	2.08-3.82	<0.001

**Table 3:** Cox proportional hazard model for depth of response.

	Unadjusted				Adjusted*		
	HR	95% CI	P		HR	95% CI	P
1-25% (n = 91)							
26-50% (n = 73)	0.58	0.42-0.80	<0.001		0.57	0.40-0.80	0.001
51-75% (n = 65)	0.49	0.35-0.69	<0.001		0.36	0.25-0.52	<0.001
76-100% (n = 36)	0.33	0.22-0.50	<0.001		0.32	0.20-0.51	<0.001

*The HR was adjusted for sex, LDH, brain metastasis.

## Abbreviations

CT: computed tomography
DepOR: depth of response
EGFR: epidermal growth factor receptor
ECOG: Eastern Cooperative Oncology Group
LDH: lactic dehydrogenase
NSCLC: no-small cell lung cancer
ORR: objective response rate
OS: overall survival
PS: performance status
PFS: progress free survival
PPS: post-progression survival
RECIST: Response Evaluation Criteria in Solid Tumors
SDs: standard deviations
TKI: tyrosine kinase inhibitors
